# High yield production of *Rhizobium* NodB chitin deacetylase and its use for *in vitro* synthesis of lipo-chitinoligosaccharide precursors

**DOI:** 10.1016/j.carres.2017.02.007

**Published:** 2017-04-10

**Authors:** Rémi Chambon, Stéphanie Pradeau, Sébastien Fort, Sylvain Cottaz, Sylvie Armand

**Affiliations:** a Univ. Grenoble Alpes, CERMAV, F-38000 Grenoble, France; b CNRS, CERMAV, F-38000 Grenoble, France

**Keywords:** Chitin, *In vitro* synthesis, Lipo-chitinoligosaccharides, NodB deacetylase, Oligosaccharides, Chitinoligosaccharide, CO, Chitinoligosaccharide deacetylated at non-reducing GlcNAc unit, CO(N), Chitin deacetylase, CD, Chitinbiose, CO-II, Chitintetraose, CO-IV, Chitinpentaose, CO-V, 1,4-dithiothreitol, DTT, D-glucosamine, GlcN, *N*-acetyl-D-glucosamine, GlcNAc, High Performance Size Exclusion Chromatography, HPSEC, Immobilized Metal ion Affinity Chromatography, IMAC, Isopropyl β-D-thiogalactopyranoside, IPTG, Lipo-chitinoligosaccharide, LCO, Matrix-Assisted Laser Desorption/Ionization Time Of Flight Mass Spectrometry, MALDI-TOF MS, Sodium dodecyl sulfate polyacrylamide gel electrophoresis, SDS-PAGE, Thin layer chromatography, TLC, Thioredoxin, Trx, Six histidine tag, His_6_ tag

## Abstract

Lipo-chitinoligosaccharides (LCOs) are key molecules for the establishment of plant-microorganisms symbiosis. Interactions of leguminous crops with nitrogen-fixing rhizobial bacteria involve Nod factors, while Myc-LCOs improve the association of most plants with arbuscular mycorrhizal fungi. Both Nod factors and Myc-LCOs are composed of a chitinoligosaccharide fatty acylated at the non-reducing end accompanied with various substituting groups. One straightforward way to access LCOs is starting from chitin hydrolysate, an abundant polysaccharide found in crustacean shells, followed by regioselective enzymatic cleavage of an acetyl group from the non-reducing end of chitin tetra- or pentaose, and subsequent chemical introduction of *N*-acyl group. In the present work, we describe the *in vitro* synthesis of LCO precursors on preparative scale. To this end, *Sinorhizobium meliloti* chitin deacetylase NodB was produced in high yield in *E. coli* as a thioredoxin fusion protein. The recombinant enzyme was expressed in soluble and catalytically active form and used as an efficient biocatalyst for *N*-deacetylation of chitin tetra- and pentaose.

## Highlights

• *Rhizobium* NodB deacetylase is expressed and purified in active form in *E. coli*.
• Yield optimization gives up to 100 mg of purified deacetylase from 1 L of culture medium.
• *In vitro* synthesis of lipo-chitinoligosaccharides precursors is performed on preparative scale.

## Graphical abstract

**Figure:**
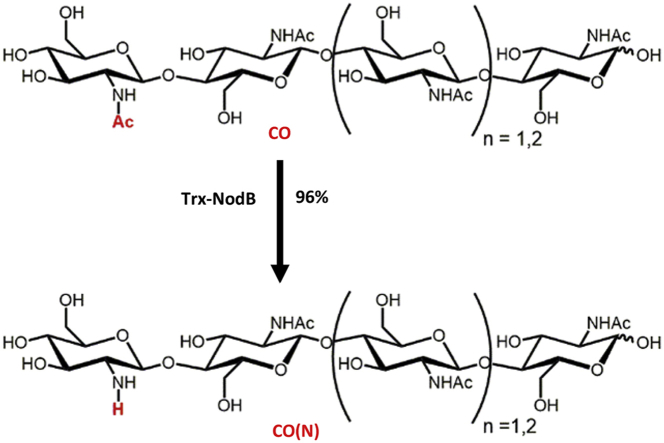

## 1 Introduction

The *Rhizobium*-legume symbiosis, formed by legume plants and rhizobial bacteria [1], promotes the assimilation of atmospheric nitrogen in legumes. Another kind of symbiosis, arbuscular mycorrhiza, is formed between a wide range of plants and mycorrhizal fungi [2]. It improves uptake of water and mineral nutrients in 80% of plant species. Both root endosymbiosis are established as a result of signals exchange in which there is mutual recognition of diffusible molecules produced by plants and microbial partners [3]. The morphogenic signal molecules produced by microsymbionts are called nodulation factors (Nod factors) for the *Rhizobium*-legume symbiosis and Myc-LCOs for the arbuscular mycorrhiza symbiosis. Nod factors and Myc-LCOs trigger profound modifications in plant-root ending intimate communications between symbionts [4].

**Fig. 1:**
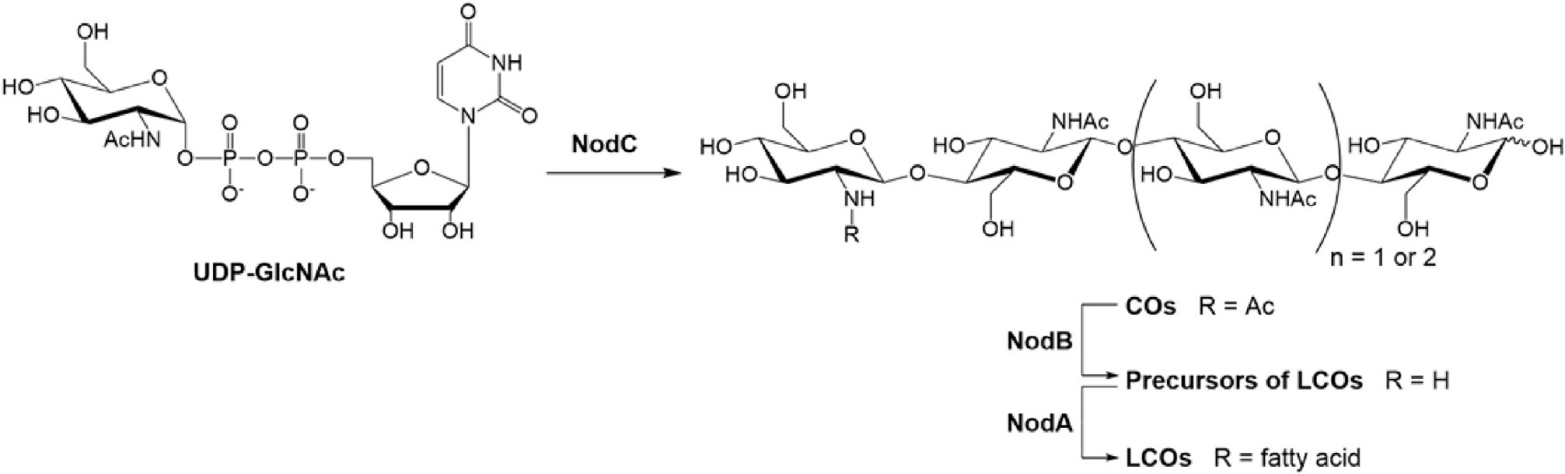
Biosynthesis pathway of core LCOs in *Rhizobium*. NodC is an *N*-acetylglucosaminyltransferase forming COs from UDP-GlcNAc; NodB is a chitin deacetylase acting solely on the non-reducing *N*-acetylglucosaminyl unit of COs; NodA is an acyltransferase acylating the free amino group from acyl protein carrier as donor.

Nod factors and Myc-LCOs are lipo-chitinoligosaccharides (LCOs). They consist of a backbone of three or four *N*-acetylglucosaminyl (GlcNAc) residues linked to an *N*-fatty acylglucosaminyl non-reducing unit together with diverse decorations (*O*-acetylation, *O*-sulfation, *O*-fucosylation, *O*-carbamoylation) on some hydroxyl groups of the reducing or non-reducing end [5]. These various substituent groups, the fatty acyl chain length and its degree of unsaturation confer their specificity in plant-host recognition [6]. LCOs are active down to nano- and even picomolar concentration, and are produced naturally in extremely small quantities [3]. Detailed recognition mechanisms of these molecules are not fully understood, activation of common symbiosis signalling pathway is thought to involve multimeric complex transmembrane receptor-like kinases (RLKs) and extracellular receptor-like proteins (LYMs) localized at the plant plasma membrane [7]. While Myc-LCOs biosynthesis pathway is still unknown, many *Rhizobium* genes are identified to be involved in Nod factors biosynthesis. Among them, three are necessary for the synthesis of the core Nod factor structures (Fig. 1).

The first step in Nod factor assembly is performed by an *N*-acetylglucosaminyltransferase encoded by *nodC* utilizing UDP-GlcNAc as substrate to give chitintetra- or pentaose (CO-IV or CO-V) [8]. Then NodB chitin deacetylase (CD) cleaves the *N*-acetyl moiety from the non-reducing terminus of the CO [9]. Finally, an acyltransferase encoded by *nodA* links the acyl chain to the resulting free amino group of the non-reducing end of the CO using an acyl carrier protein as a donor [10].

Based on sequence similarities studies [11], NodB CD belongs to the carbohydrate esterase CE-4 family, including four major members that share a conserved region in the primary structure assigned as the NodB homology domain [12]: peptidoglycan GlcNAc deacetylases and peptidoglycan *N*-acetylmuramic acid deacetylases (EC 3.5.1.–) play a role in the modification of the bacterial cell wall; acetylxylan esterases (EC 3.1.1.72) catalyse the removal of acetyl groups from acetylated xylan and xylo-oligosaccharides; CDs (EC 3.5.1.41) catalyse the hydrolysis of GlcNAc acetamido group in chitin and chitin oligomers. CDs are expressed in several fungi and insects and are committed in the synthesis and the morphogenesis of cell walls [13]. CDs are also present in marine bacteria and archaea, being involved in chitin degradation [14], [15].

COs and their derivatives take part in landmark recognition molecular events whose Nod and Myc-factors are a compelling example. Biological activities of COs are dependent on CO size and N-acetylation pattern [16], [17], highlighting the action of CDs. Despite their affiliation to the CE-4 family, CDs have diverse modes of action, ranging from a single to multiple attack depending on CO length and displaying various regioselectivity. NodB CD displays a stringent regioselectivity releasing acetic acid solely from the non-reducing end of COs.

In the first report describing the production of recombinant *Sinorhizobium meliloti* NodB in *E. coli*, the enzyme accumulated in the form of non-functional insoluble aggregates called inclusion bodies [9]. While expression as inclusion body can sometimes be advantageous, the *in vitro* refolding is an empirical process and there is no guarantee that it will lead to large amount of active enzyme. Recently, *Rhizobium* sp. GHR2 NodB was expressed and purified under soluble and enzymatically active form in *E. coli*, which opens the way to the production of recombinant NodB without the need of refolding it [17]. The objective of this work was to develop an optimized process for the preparative scale *in vitro* synthesis of LCO precursors from biosourced chitin oligomers. To this end, *Sinorhizobium meliloti* NodB was produced in high yield in *E. coli* and the recombinant enzyme expressed in a soluble and catalytically active form was then efficiently used as biocatalyst.

## 2 Materials and methods

### 2.1 General

Reagents for bacterial media were from Euromedex (Mundolsheim, France) and Invitrogen (Cergy-Pontoise, France). Reagents for molecular biology were obtained from Invitrogen, Euromedex, Macherey-Nagel (Hoerd, France) and Thermo Fisher Scientific (Villebon sur Yvette, France). Oligonucleotides were purchased from Eurofins MWG Operon (Germany). FACOS™ (a low molecular weight chitosan inferior to 2000 g mol^−1^ with an acetylation degree of 10%) was purchased from Kitto Life Co (Kyongki-Do, South Korea). Chemicals were purchased from Sigma-Aldrich Chimie (Saint Quentin-Fallavier, France). The mass measurements of chitinoligosaccharides were performed using a MALDI-ToF/ToF AutoFlex I (Bruker) at PSM facility, PCN-ICMG, Grenoble.

### 2.2 Cloning constructs for NodB expression in *Escherichia coli*

*Sinorhizobium meliloti nodB* gene was amplified by PCR using forward (5′-GGCCATGGAGCACCTCGATTAC-3′) and reverse (5′-CCTCGAGGTGATGCGGAGGAAG-3′) primers containing respectively a *Nco*I site and a *Xho*I site (underlined). The PCR product was digested with *Nco*I and *Xho*I restriction enzymes and cloned in-frame with a six histidine tag (His_6_ tag) into pET21d vector to produce the pET21d-nodB expression plasmid that encodes the NodB-His_6_ fusion protein. Alternatively, the digested PCR product was cloned in-frame with the thioredoxin *trxA* gene into pET32a vector, generating pET32a-nodB expression plasmid that encodes the Trx-NodB fusion protein.

### 2.3 Expression and purification of recombinant NodB CD

*E. coli* strain BL21 (DE3) harbouring pET21d-nodB or pET32a-nodB plasmid was cultured at 37 °C to mid-exponential phase (to a cell density of 0.6 at 600 nm) in Luria broth supplemented with 100 μg mL^−1^ ampicillin under constant shaking (180 rpm). Isopropyl β-D-thiogalactopyranoside (IPTG) was then added at a final concentration of 0.5 mM to induce recombinant gene expression, and the culture incubated for a further 22 h at 16 °C. Cells were then harvested by centrifugation at 8450 g for 10 min at 4 °C, resuspended in equilibration buffer (20 mM Tris-HCl pH 8, 500 mM NaCl, 20 mM imidazole) and lysed with a Cell Disruption System (Constant Systems Ltd) at 1.9 kbars. After centrifugation at 50,000 g for 30 min at 4 °C, the supernatant (crude cytoplasmic extract) was submitted to Immobilized Metal ion Affinity Chromatography (IMAC) using a Ni^2+^-nitrilotriacetate-agarose resin (Qiagen). Recombinant NodB fusion proteins were allowed to bind to the matrix, and after washing with the equilibration buffer, they were eluted with a 20–300 mM imidazole gradient in the same buffer. All elution fractions were analysed by SDS-PAGE. Fractions containing fusion proteins were dialyzed against 20 mM MOPS buffer pH 7.2 and stored at - 80 °C until further use. The protein concentration was determined by the Bradford method with BSA as a standard [18].

#### 2.3.1 SDS-PAGE

Protein samples were heated at 100 °C for 5 min in Laemmli buffer [19] and analysed by SDS-PAGE on a 12% polyacrylamide gel, followed by staining with Instant Blue (Expedeon).

### 2.4 Enzyme assays

NodB CD activity was assayed by incubating CO-II or CO-V with 0.35 μM CD in 20 mM MOPS pH 7.2 containing 10 mM DTT and 1 mM MnSO_4_ at 30 °C. At regular time intervals, the amount of released amino groups was quantified by using 3-methyl-2-benzothiazolinone hydrazone hydrochloride reagent (MBTH) [20]. For the determination of kinetic parameters, measurements of initial rates were made at several substrate concentrations ranging from 0.2 to 4.5 mM. *K*_M_ and *k*_cat_ were determined by non-linear regression using the Grafit program (Erithacus Software).

### 2.5 Preparation of COs

Commercial chitosan oligosaccharides (4 g, FACOS™) were *N*-acetylated by treatment with acetic anhydride (40 mL) and triethylamine (60 mL) in water-methanol (1:1 v/v) (600 mL) for 12 h at 20 °C followed by concentration *in vacuo*. The resulting solid was dissolved in water and purified through a cationic exchange resin DOWEX 50WX4 (H^+^ form) column to eliminate the partially acetylated oligosaccharides using water as eluent. The non-retained fraction containing COs was neutralized with an anionic exchange resin Amberlite IRA400 (OH^−^ form) and freeze-dried. COs were individually separated by size exclusion chromatography (SEC) consisting of a Biogel P4 colomn (1.5 × 1000 mm) connected to a pump (Smartline semi-preparative, Knauer) and with an inline degasser (Knauer). Elution was performed with water at a flow rate of 2.25 mL min^−1^, and monitored by a refractometric detector (IOTA-2, Precision Instrument (DIONEX)). COs homogeneity was confirmed by MALDI-TOF MS and HPSEC analyses [21].

### 2.6 CD catalyzed deacetylation of COs

Chitinoligosaccharide (CO-IV or CO-V) (15 mg) was incubated with 750 μg of NodB CD in 20 mM MOPS pH 7.2 (5 mL) containing 10 mM DTT and 1 mM MnSO_4_ during 20 h at 30 °C. The enzyme was heat-inactivated at 100 °C for 10 min, and the reaction mixture was centrifuged for 10 min at 21 100 g at 4 °C. The resulting supernatant was freeze-dried and re-suspended in a mixture of water and acetone (1/20). The white precipitate corresponding to purified CO-IV(N) or CO-V(N) was characterized by MALDI-TOF MS and HPSEC, and was compared to authentic samples [22], [23].

#### 2.6.1 Thin layer chromatography

Reactions were monitored by thin-layer chromatography (TLC) using Silica Gel 60 F254 precoated plates (E. Merck, Darmstadt) and propanol/H_2_O/ammonia (70:30:1) as a solvent. Detection of carbohydrates was achieved by charring with 30% ammonium bisulfate.

#### 2.6.2 Analytical size-exclusion chromatography (HPSEC)

COs and CO(N)s were analysed by HPSEC. The system consisted of a Shodex OH pack SB-G precolumn in front of a series of a Shodex OH pack SB-802 and a Shodex OH pack SB-802.5 columns (8*300 mm), connected to a pump (Waters 510, Millipore). Samples were solubilized in water. Elution was performed with a 100 mM sodium nitrate solution at a flow rate of 0.5 mL min^−1^ and monitored by a refractometric detector (Waters 410, Millipore).

## 3 Results and discussion

### 3.1 Expression and purification of NodB-His_6_ CD

**Fig. 2:**
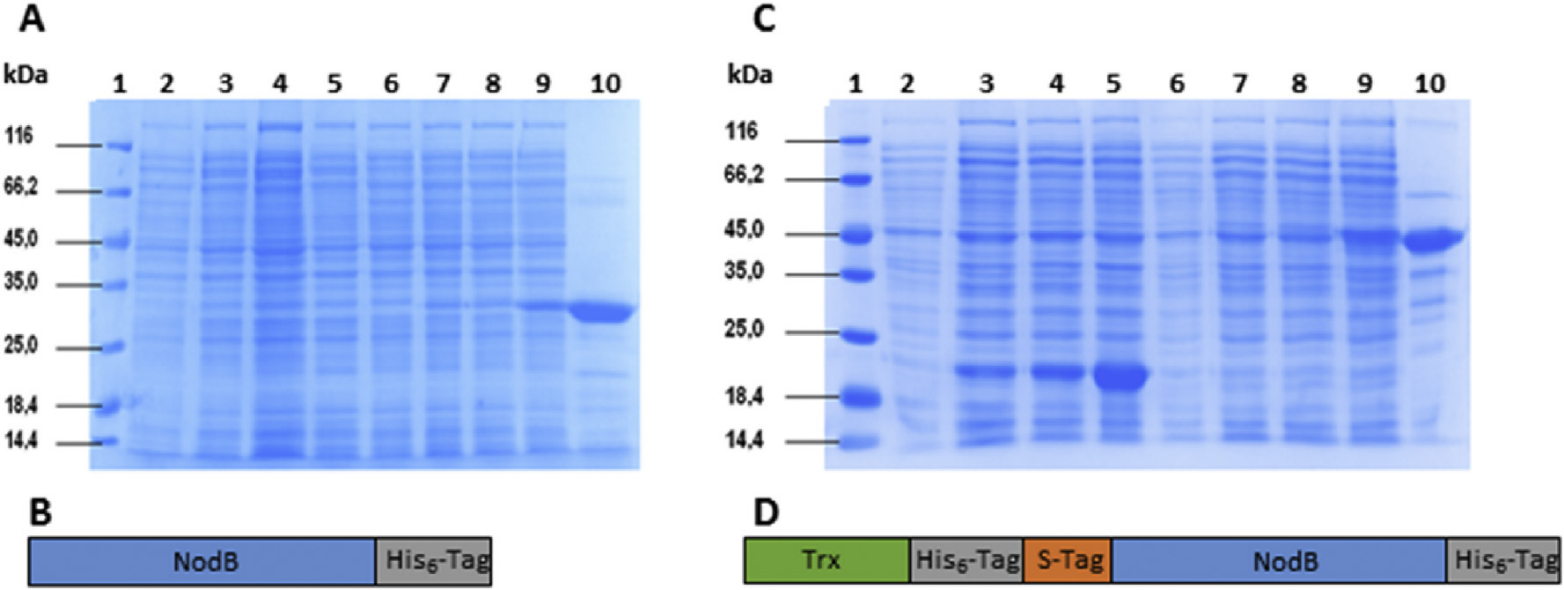
Heterologous expression of recombinant NodB CD in *E. coli* BL21 (DE3) analysed by 12% SDS-PAGE. **(A)** Lane 1, molecular weight marker; lanes 2–5 show protein patterns of cell lysates of bacteria transformed by pET21d obtained after IPTG induction for 0, 2, 6 and 22 h; lanes 6–9, show protein patterns of cell lysates of bacteria transformed by pET21d-nodB obtained after IPTG induction for 0, 2, 6 and 22 h; lane 10 shows NodB-His_6_ CD after IMAC purification. **(B)** Schematic representation of NodB-His_6_ CD. The pET21-nodB plasmid encodes a C-terminal six histidine tag fused to NodB (the figure is not to scale). **(C)**. Lane 1, molecular weight marker; lanes 2–5 show protein patterns of cell lysates of bacteria transformed by pET32a obtained after IPTG induction for 0, 2, 6 and 22 h; lanes 6–9, show protein patterns of cell lysates of bacteria transformed by pET32a-nodB obtained after IPTG induction for 0, 2, 6 and 22 h; lane 10 shows Trx-NodB CD after IMAC purification. **(D)** Schematic representation of Trx-NodB CD. The pET32a-nodB plasmid encodes a fusion protein containing a Trx tag followed by an additional 52 amino acids sequence containing His_6_ and S-tag at the N-terminus of NodB, and a second His_6_ tag at the C-terminus (the figure is not to scale).

To our knowledge, only two reports describe the overexpression of NodB CD in *E. coli*. In the first one, *Sinorhizobium meliloti* NodB CD was produced as inactive protein aggregates from which the enzyme could be refolded [9]. In the second one, *Rhizobium* sp. GRH2 NodB CD was expressed as an active soluble enzyme [17]. In both case, expression levels were not shown, and large-scale overexpression of NodB CD still remains challenging. *Sinorhizobium meliloti nodB* gene was first cloned into pET21d vector. The resulting overexpression pET21d-nodB plasmid encoded an additional sequence of eight amino acids (LEHHHHHH) at the C-terminus in comparison to the wild type protein (Fig. 2B). The construct was introduced into *Escherichia coli* BL21 (DE3) expression strain by transformation. In this plasmid, the expression is under the control of T7 promoter. Optimal expression conditions were established by varying the incubation time and IPTG concentration (0.05, 0.1 and 0.5 mM), while induction temperature was kept at 16 °C. Using the optimized conditions (induction with 0.5 mM IPTG during 22 h), NodB soluble overexpression could be observed (Fig. 2A, lane 9), but together with inclusion bodies (data not shown). Bacteria transformed by pET21d alone did not show any overexpression in the same conditions (Fig. 2A, lanes 2–5). The overexpressed NodB-His_6_ was purified from crude cytoplasmic extract by IMAC on a Ni^2+^ resin yielding 15 mg of 85% pure fusion protein per litre of culture (Fig. 2A, lane 10). The 30 kDa apparent molecular weight of NodB-His_6_ on SDS-PAGE was in agreement with the expected size of 28.5 kDa [9]. This fusion protein proved to be active when assayed on CO-II as substrate (data not shown).

### 3.2 Expression and purification of Trx-NodB CD

To explore the possibility of improving expression level, *Sinorhizobium meliloti nodB* gene was then cloned into pET32a vector which contains *E. coli* thioredoxin (*trxA*) as a gene fusion partner. Thioredoxin is a 109 amino acids protein known to increase the solubility and yield of heterologous proteins synthesized in the *E. coli* cytoplasm [24]. The pET32a-nodB plasmid encodes a fusion protein containing the Trx tag followed by a His_6_ tag and an S-tag at the N-terminus of NodB, and an additional His_6_ tag at the C-Terminus (Fig. 2D). For clarity, this fusion protein will be called Trx-NodB. While *E. coli* BL21 (DE3) harbouring pET32a only overexpressed thioredoxin under the optimized induction conditions set before (Fig. 2C, lanes 2–5), bacteria transformed with pET32a-nodB plasmid overexpressed a 42 kDa recombinant protein, in good agreement with the calculated molecular weight of 41.9 kDa for Trx-NodB (Fig. 2C, lanes 6–9). IMAC purification of Trx-NodB from crude cytoplasmic extract gave 100 mg of 73% pure fusion protein per litre of culture. Regarding molar ratios, Trx-NodB was overexpressed at a yield fourfold higher than NodB-His_6_. Trx-NodB fusion protein was found to be stable for several months upon storage at - 80 °C without addition of glycerol or other stabilizer. It was used to performed biochemical characterization and preparation of precursors of LCOs.

### 3.3 Enzyme assays and biochemical characterization

**Fig. 3:**
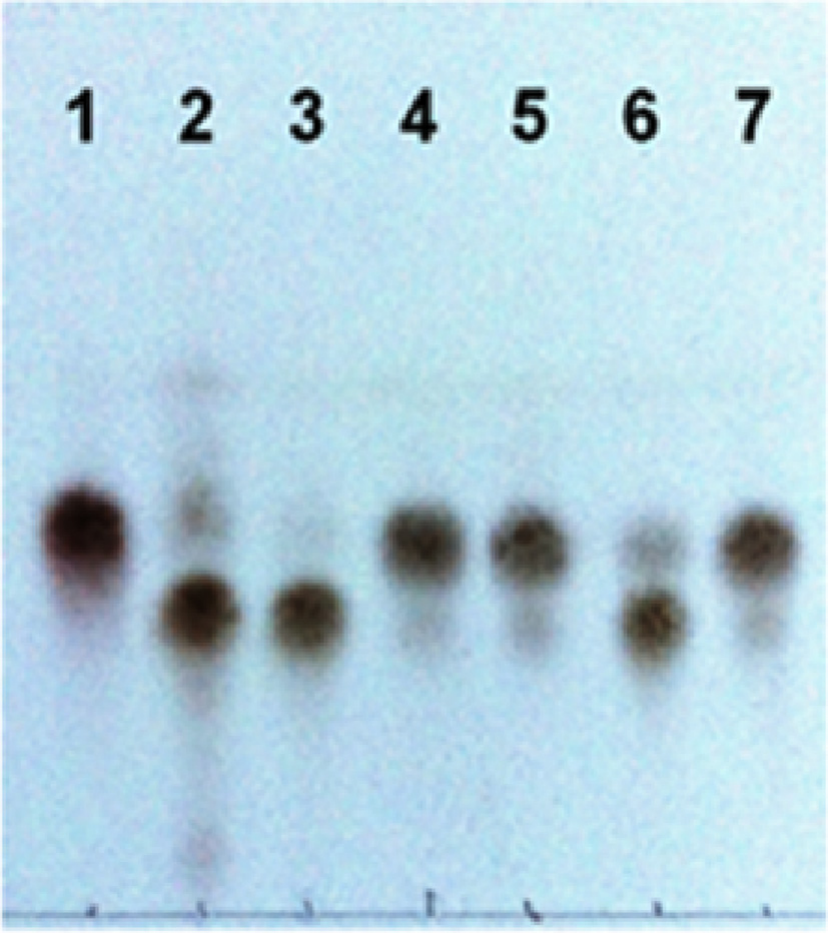
Thin layer chromatography analysis of NodB CD activity on CO-V in presence of divalent metal cation and reducing agent. Lane 1, CO-V; lane 2, CO-V(N); NodB activity on CO-V with: lane 3, DTT + MnSO_4_; lane 4, MnSO_4_; lane 5, DTT; lane 6, DTT + MgSO_4_; lane 7, DTT + ZnSO_4_. DTT was at a concentration of 10 mM, divalent metal cation at a concentration of 1 mM, in 20 mM MOPS pH 7.2.

*S. meliloti* NodB CD is known to have an optimum activity between pH 7 and 8 at 30 °C [9]. MOPS (20 mM pH 7.2) was the preferred buffer for this study, while NodB was not active in ammonium bicarbonate buffer in contrast with recombinant NodB from *Rhizobium* sp. GRH2 [17]. Most members of CE4 family are metalloenzymes possessing a divalent metal cation acting as cofactor and necessary for activity [25]. Although recombinant *S. meliloti* NodB CD was catalytically competent without the need of adding such cation [9], we found Trx-NodB CD inactive under these conditions. This was probably due to interference with Ni^2+^-chelating resin during IMAC purification and removal of the metal originally present in the recombinant protein. The capability to restore enzymatic activity of Trx-NodB CD was evaluated with several divalent cations. Among them Mn^2+^ and Mg^2+^ proved to be the most active (Fig. 3), while Zn^2+^, Co^2+^, Cu^2+^, Fe^2+^, Cd^2+^ and Ni^2+^ did not give significant results. This last finding was unexpected since Zn^2+^ and Co^2+^ are usually the preferred metal for CE4 family enzymes, being activators for various CD from *Mucor rouxii* [26], *Cellulotricum lindemuthianum* [27] or *Vibrio cholerea* [25]. DTT was also necessary for Trx-NodB activity, indicating that active CD is probably on reduced form (Fig. 3).

Kinetic properties of Trx-NodB CD are illustrated in Table 1. Trx-NodB CD exhibited no detectable activity on monosaccharide GlcNAc as already observed with untagged NodB and NodB-Strep-tag [9], [17], and was active on COs ranging from chitinbiose (CO-II) to chitinhexaose (CO-VI) (data not shown). Kinetics with CO-II and CO-V chosen as model substrates showed Michaelis-Menten behaviour. Since most Nod factors consist of a backbone of three, four or five β-1,4-linked *N*-acetylglucosaminyl residues *N*-acylated at the non-reducing end [5], CO-V can be considered as one of the natural substrate for *S. meliloti* NodB CD. Not surprisingly, it is a better substrate than CO-II, with a catalytic efficiency fivefold superior for the pentamer compared to the dimer substrate. Interestingly, the turnover number *k*_cat_ is comparable for the two substrates, while *K*_M_ for CO-V is four times lower than for CO-II, indicating that the active site is better adapted for longer substrates. This observation may be related to the active site of *Vibrio cholerae* 1280 CD (VC1280) which shares 18.3% identity with NodB. VC1280 is the only CD for which the structure of the active site spanned with a CO ligand is known to date [25]. It possesses three subsites (−2, −1 and 0), and chitinbiose (CO-II) is the best substrate (definition and nomenclature for the numbering of sugar-binding subsites in Ref. [28]). It is tempting to speculate that *S. meliloti* NodB CD may harbour a larger number of subsites, explaining the better affinity for CO-V. We have shown that pure NodB can be produced in sufficient amount to envisage its crystallization. We can hope that crystal structure of NodB in complex with its natural substrate will help to confirm this hypothesis and lead to a better understanding of the mechanism which governs the regioselectiviy of both CDs. The catalytic efficiency between the two CDs are strikingly similar and *V. cholerae* 1280 CD have proved to be a powerful catalyst for *in vitro* synthesis of analogues of LCO precursors, *N*-deacetylated on the penultimate unit of CO [21]. There is therefore good hope that Trx-NodB CD would be a suitable catalyst for the preparative scale synthesis of LCO precursors (*N*-deacetylated on the non-reducing end) from chitin oligomers.

### 3.4 CD catalyzed deacetylation of COs

Having developed satisfactory conditions for the production of soluble and catalytically active NodB CD, the next step was to design an effective process for the conversion of COs on preparative scale. When CO-IV or CO-V was incubated with Trx-NodB in a molar ratio of 1/1000, TLC analysis indicated the quantitative transformation in deacetylated products after 20 h of reaction. This shows that Trx-NodB is stable and not inhibited by the release of acetic acid under these conditions. Optimization of the isolated yield was achieved by establishing a simple purification procedure. The reaction mixture was freeze-dried after heat inactivation and filtration of the denatured protein, and the solid was solubilized in a minimum of H_2_O before precipitation in acetone giving deacetylated CO in high purity (Table 2). The precipitate contained no remaining buffer and no starting CO as judged by MALDI-MS and HPSEC analyses. CO-IV(N) was obtained in 84% isolated yield, slightly contaminated with CO-V(N) originated from residual CO-V present in starting CO-IV (Fig. S1a–b). CO-V(N) was isolated in high yield (90%) and high purity as indicated by HPSEC and MALDI-MS analyses (Fig. S1c–d). The reproducibility of the reaction was evaluated by performing 10 times the reaction starting from 100 mg of CO-V. The result was excellent with an average yield of 92% over all experiments (Table 2).

## 4 Conclusions

In summary recombinant *S. meliloti* NodB CD can be efficiently produced and purified from *E. coli* as a thioredoxin fusion protein, under soluble and catalytically active form. Trx-NodB CD is suitable for *in vitro* conversion of CO regioselectively deacetylated at the non-reducing end, and the procedure has been validated for the synthesis of precursors of LCO at 100 mg-scale. In regards to the Nod factors and Myc-LCO concentration inducing responses in plants, this approach constitutes a real breakthrough concerning the production of high value molecules for sustainable agriculture.

## Authors' contribution

Rémi Chambon - cloning, expression, purification, biochemical studies of CD, enzymatic synthesis, article preparation; Stéphanie Pradeau - preparation of COs; Sébastien Fort - planning of experiments; Sylvain Cottaz - planning of experiments, article preparation; Sylvie Armand - planning of experiments, article preparation.

## Figures and Tables

**Table 1:** Kinetic parameters of Trx-NodB CD.

Substrate	*K*_M_ (μM)	*k*_cat_ (s^−1^)	*k*_cat_/*K*m (s^−1^ M^−1^)
CO-II	1530 ± 200	0.38 ± 0.03	248.6 ± 17.2
CO-V	379 ± 23	0.45 ± 0.05	1172 ± 48

NodB CD activity was assayed by incubating CO-II or CO-V with 0.35 μM CD in 20 mM MOPS pH 7.2 containing 10 mM DTT and 1 mM MnSO_4_ at 30 °C. Measurements of initial rates were made at several substrate concentrations ranging from 0.2 to 4.5 mM. *K*_M_ and *k*_cat_ were determined by non-linear regression.

**Table 2:** Enzymatic deacetylation of COs at preparative scale.

Starting compound	Quantity (mg/μmol)	Product	Quantity (mg/μmol)	Isolated yield (%)
CO-IV	15/18	CO-IV(N)	12/15.2	84
CO-V	15/14.5	CO-V(N)	13/13.1	90
CO-V	100/96.8	CO-V(N)	89/89.8^a^	92^a^

COs were incubated with Trx-NodB CD in 20 mM MOPS pH 7.2 containing 10 mM DTT and 1 mM MnSO_4_ during 20 h at 30 °C, followed by purification by precipitation in water/acetone. 750 μg (17.85 nmol) of Trx-NodB and 5 mL of buffer were used for experiments at 15 mg scale. 4800–7200 μg (114–171 nmol) of Trx-NodB and 39 mL of buffer were used for experiments at 100 mg scale.

a Isolated yield for procedure at 100 mg scale is averaged over 10 experiments.
