# Supplementary material for: High yield production of Rhizobium NodB chitin deacetylase and its use for in vitro synthesis of lipo-chitinoligosaccharide precursors
Source: Carbohydr Res. 2017 Apr 10;442:25–30. doi: 10.1016/j.carres.2017.02.007 (PMC5380657; doi:10.1016/j.carres.2017.02.007)
Supplement: Supplementary file 1 [file mmc1.docx]

**Supporting information**


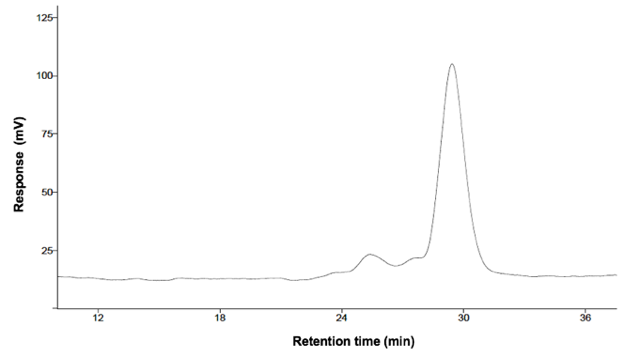


Fig. S1a. Analytical size-exclusion chromatography of CO-IV(N) after purification. The sample was solubilized in water and elution was performed with 100 mM sodium nitrate.


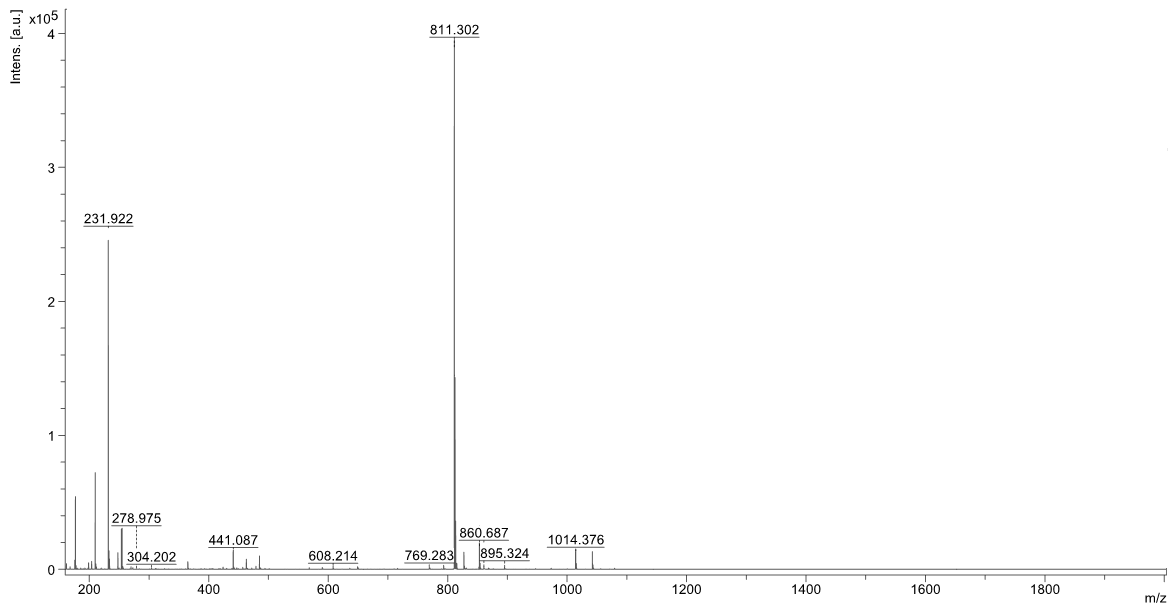


Fig. S1b. Low resolution MALDI-TOF spectrum of CO-IV(N) after purification.


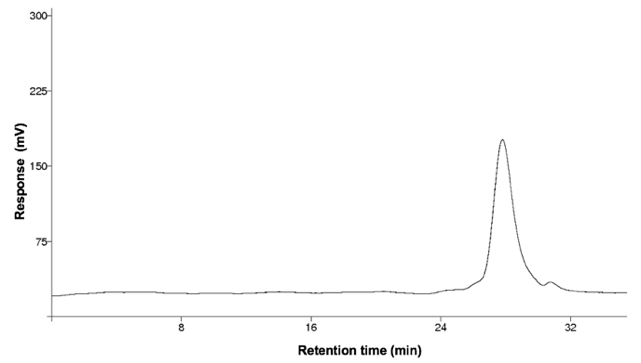


Fig. S1c. Analytical size-exclusion chromatography of CO-V(N) after purification. The sample was solubilized in water and elution was performed with 100 mM sodium nitrate.


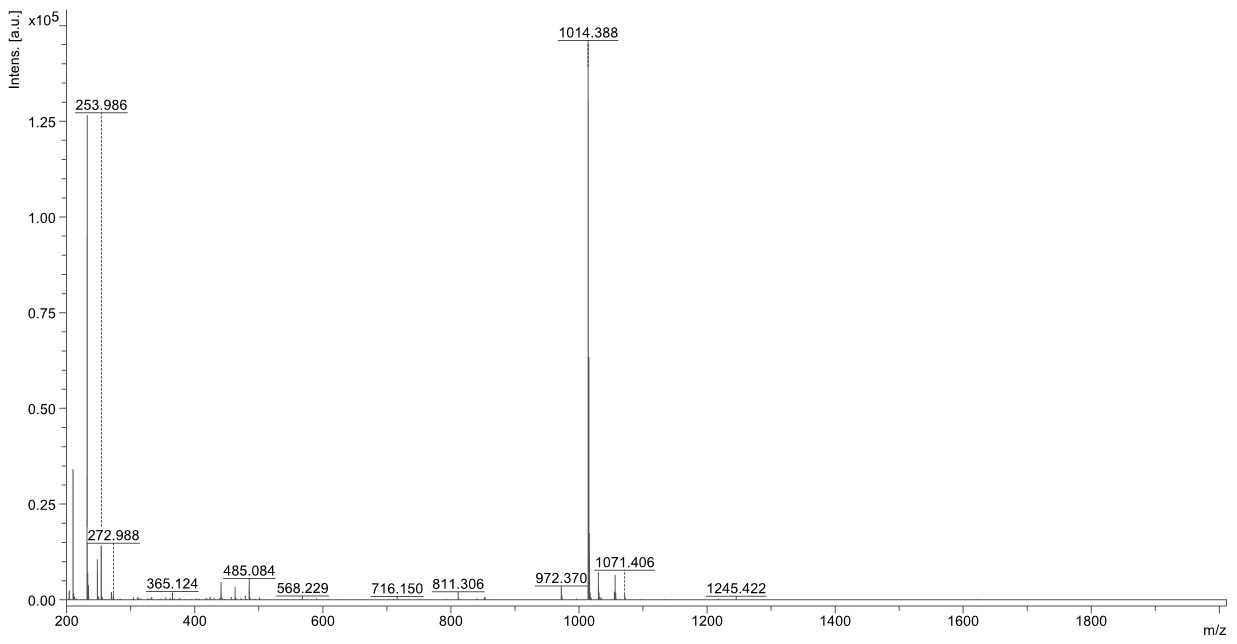


**Fig. S1d.** Low resolution MALDI-TOF spectrum of CO-V(N) after purification.
